# Quality and user satisfaction scores for prosthetic limbs provided in a fitting camp, Cambodia

**DOI:** 10.2471/BLT.25.294638

**Published:** 2025-10-20

**Authors:** Thearith Heang, Sisary Kheng, Maggie Donovan-Hall, Amos Channon, Alex Dickinson, Carson Harte

**Affiliations:** a Exceed Cambodia, Phnom Penh, Cambodia.; b School of Health Sciences, University of Southampton, Southampton, England.; c Department of Social Statistics and Demography, University of Southampton, Southampton, England.; d School of Engineering, University of Southampton, Southampton, England.; e Exceed Worldwide, Lisburn Square, Lisburn, BT28 1TS, Northern Ireland.

## Abstract

**Objective:**

To evaluate prosthetic devices delivered during an intensive fitting camp in Cambodia, considering device quality and client satisfaction.

**Methods:**

We conducted an observational cohort study in which we assessed prosthetic devices produced and delivered by an international nongovernmental organization at an intensive fitting camp. We conducted our assessment in two stages: at prosthetic device provision using a checklist to assess device quality and client satisfaction at discharge, and at 3-month follow-up using a telephone interview to assess the client’s device usage and preference for a future device.

**Findings:**

We found that many of the devices fitted at the camp failed to meet International Society for Prosthetics and Orthotics’ standards. Assessments revealed dissatisfaction with quality of work (33%; 175/525), fit (57%; 297/525) and function (26%; 139/525). At follow-up, 36% of clients (115/321) reported discomfort or pain. Most clients (78%; 238/305) stated preference for a domestically-produced device in future. Most clients (81%; 253/313) reported using their new device not very often, rarely or never, whereas 88% (243/277) of clients with a previous device reported using that device often or always. At least 29% (93/321) continued to use a previous device that they had described during the camp as unused, broken, painful or poorly fitting.

**Conclusion:**

Our findings indicate that shortcomings in quality and satisfaction of the studied prosthesis system persist as many clients rely on an inadequate or potentially dangerous prosthesis. The findings also raise new questions about client selection and the effective use of funding for the intensive camp provision format.

## Résumé

**Objectif:**

Évaluer les prothèses fournies lors d’un camp d’appareillage intensif au Cambodge, en tenant compte de la qualité des prothèses et de la satisfaction des clients.

**Méthodes:**

Nous avons mené une étude de cohorte observationnelle pour évaluer les prothèses produites et fournies par une organisation non gouvernementale internationale lors d’un camp d’appareillage intensif. Nous avons mené notre évaluation en deux étapes: lors de la fourniture des prothèses, à l’aide d’une liste de contrôle pour évaluer la qualité des prothèses et la satisfaction des clients à leur sortie, et lors d’un suivi à trois mois, à partir d’un entretien téléphonique pour évaluer l’utilisation des prothèses par les clients et leurs préférences pour une éventuelle future prothèse.

**Résultats:**

Nous avons constaté que de nombreuses prothèses posées lors du camp ne répondaient pas aux normes de la Société internationale de prothèse et orthèse . Les évaluations ont révélé une insatisfaction concernant la facture des prothèses (33%; 175/525), leur ajustement (57%; 297/525) et leur fonctionnement (26%; 139/525). Lors du suivi, 36% des clients (115/321) ont signalé une gêne ou une douleur. La plupart d’entre eux (78%; 238/305) ont déclaré préférer une prothèse fabriquée localement à l’avenir. La plupart (81%; 253/313) ont déclaré utiliser leur nouvelle prothèse peu souvent, rarement ou jamais, tandis que 88% (243/277) des clients possédant une ancienne prothèse ont déclaré l’utiliser souvent ou toujours. Au moins 29% (93/321) ont continué à utiliser une ancienne prothèse qu’ils avaient décrite auparavant (pendant le camp) comme inutilisée, cassée, provoquant des douleurs ou mal ajustée.

**Conclusion:**

Nos résultats indiquent que les lacunes en matière de qualité et de satisfaction du système d’appareillage étudié persistent, car de nombreux clients dépendent d’une prothèse inadéquate ou potentiellement dangereuse. Les résultats soulèvent également de nouvelles questions concernant la sélection des clients et l’utilisation efficace des fonds pour le format de prestation intensive lors du camp.

## Resumen

**Objetivo:**

Evaluar las prótesis suministradas durante un campamento intensivo de adaptación en Camboya, considerando la calidad de los dispositivos y la satisfacción de los usuarios.

**Métodos:**

Se realizó un estudio de cohorte observacional en el que se evaluaron las prótesis producidas y entregadas por una organización no gubernamental internacional en un campamento intensivo de adaptación. La evaluación se realizó en dos fases: en el momento de la entrega de la prótesis, mediante una lista de verificación para valorar la calidad del dispositivo y la satisfacción del usuario al alta, y a los tres meses de seguimiento, mediante una entrevista telefónica para conocer el uso del dispositivo y la preferencia de los usuarios respecto a futuras prótesis.

**Resultados:**

Se observó que muchas de las prótesis adaptadas durante el campamento no cumplían las normas de la Sociedad Internacional de Prótesis y Órtesis. Las evaluaciones revelaron insatisfacción con la calidad de la fabricación (33%; 175/525), el ajuste (57%; 297/525) y la función (26%; 139/525). En el seguimiento, el 36% de los usuarios (115/321) refirió malestar o dolor. La mayoría (78%; 238/305) manifestó preferir en el futuro una prótesis de fabricación nacional. Además, el 81% (253/313) informó usar su nueva prótesis con poca frecuencia, raramente o nunca, mientras que el 88% (243/277) de quienes disponían de una prótesis anterior declaró usarla a menudo o siempre. Al menos el 29% (93/321) continuó utilizando una prótesis previa que, durante el campamento, había descrito como inutilizada, rota, dolorosa o mal ajustada.

**Conclusión:**

Los resultados indican que persisten deficiencias en la calidad y la satisfacción de los usuarios del sistema protésico evaluado, ya que muchos siguen dependiendo de prótesis inadecuadas o potencialmente peligrosas. Los resultados también plantean nuevos interrogantes sobre la selección de los usuarios y la eficacia del uso de los recursos destinados al formato de campamentos intensivos de adaptación protésica.

## ملخص

الغرض تقييم الأجهزة الاصطناعية المُقدمة خلال معسكر تركيب مكثف في كمبوديا، مع مراعاة جودة الجهاز ورضا العملاء.

الطريقة قمنا بإجراء دراسة رصدية جماعية، حيث قمنا بتقييم الأجهزة الاصطناعية التي أنتجتها وسلمتها منظمة دولية غير حكومية في معسكر تركيب مكثف. قمنا بإجراء التقييم على مرحلتين: عند توفير الأجهزة الاصطناعية باستخدام قائمة مرجعية لتقييم جودة الجهاز ورضا العملاء عند الخروج من المستشفى، وعند متابعة بعد 3 أشهر عبر مقابلة هاتفية لتقييم استخدام العميل للجهاز وتفضيله لجهاز آخر في المستقبل.

النتائج وجدنا أن العديد من الأجهزة التي تم تركيبها في المعسكر لم تستوفِ معايير الجمعية الدولية للأطراف الاصطناعية وتقويم العظام. كشفت التقييمات عن عدم الرضا بخصوص جودة الصنع (%33؛ 175/525)، والملاءمة (%57؛ 297/525)، والوظيفة (%26؛ 139/525). عند المتابعة، أبلغ %36 من العملاء (115/321) عن شعورهم بعدم الراحة أو الألم. أوضح معظم العملاء (%78؛ 238/305) تفضيلهم لجهاز محلي الصنع في المستقبل. أبلغ معظم العملاء (%81؛ 253/313) عن استخدامهم لأجهزتهم الجديدة بشكل غير متكرر، أو نادر، أو عدم الاستخدام مطلقًا، بينما أعلن %88 (243/277) من العملاء الذين لديهم جهاز سابق، عن استخدامهم هذا الجهاز كثيرًا أو دائمًا. استمر ما لا يقل عن %29 (93/321) في استخدام جهاز سابق وصفوه سابقًا (أثناء المعسكر) بأنه غير مستخدم، أو مكسور، أو مؤلم، أو غير مناسب.

الاستنتاج تشير نتائجنا إلى أن أوجه القصور في الجودة والرضا المتعلقة بنظام الأطراف الاصطناعية المدروس، لا تزال قائمة حيث يعتمد العديد من العملاء على أطراف اصطناعية غير مناسبة أو يحتمل أن تكون خطرة. كما تثير النتائج أسئلة جديدة حول اختيار العملاء، والاستخدام الفعال للتمويل لتنسيق الإمداد في المعسكر المكثف.

## 摘要

**目的:**

旨在评估柬埔寨某个密集型假肢适配营地内交付的假肢装置的质量和用户满意度。

**方法:**

我们开展了一项观察性队列研究,在研究期间,我们评估了某一国际非政府组织在某个密集型假肢适配营地处生产和交付的假肢装置。我们分别在以下两个阶段开展了评估工作:在假肢装置交付阶段,利用检查单来评估假肢装置的质量和用户出院时的满意度;在为期 3 个月的后续随访阶段,通过开展电话调查来评估用户的假肢装置使用情况以及对未来假肢装置的偏好。

**结果:**

我们发现,在该适配营地装配的很多假肢装置均不符合国际假肢矫形学会的相关标准。评估结果显示,对假肢装置的工艺、适配度和功能感到不满意的用户所占比例分别为 33% (175/525)、57% (297/525) 和 26% (139/525)。在后续随访阶段,36% 的用户 (115/321) 报告其出现了不适或疼痛现象。大多数用户(78%,238/305)表示其未来更倾向于选择国产假肢装置。大多数用户(81%,253/313)报告其不常、很少或从未使用过其新假肢装置,然而 88% (243/277) 拥有旧假肢装置的用户称其经常或总是使用其旧装置。至少有 29% (93/321) 的用户继续使用其之前(在适配营地内)声称从未使用过、已损坏、会引发疼痛或适配度极低的旧假肢装置。

**结论:**

鉴于很多用户声称其所用假肢质量不够好或存在潜在危险,研究结果表明,我们研究的假肢系统始终存在质量缺陷和用户满意度较低的问题。根据研究结果和针对密集型假肢适配营地交付方式,我们还发现了一系列涉及用户选择和资金有效利用的新问题。

## Резюме

**Цель:**

Оценить протезные устройства, предоставленные в ходе интенсивной программы подгонки на базе временного пункта протезирования в Камбодже, с точки зрения качества устройств и удовлетворенности клиентов.

**Методы:**

Авторы провели обсервационное когортное исследование, в рамках которого оценили протезные устройства, изготовленные и предоставленные международной неправительственной организацией в ходе интенсивной программы на базе временного пункта протезирования. Оценка проводилась в два этапа: при предоставлении изделия его качество и удовлетворенность пациента в момент выписки оценивали с помощью контрольного опросного листа, а три месяца спустя контроль осуществлялся путем опроса по телефону с оценкой использования клиентом полученного устройства и его предпочтений в отношении подобных устройств в будущем.

**Результаты:**

Было обнаружено, что многие устройства, подогнанные во временном пункте, не отвечали стандартам Международного общества протезирования и ортопедии. В ходе оценки было выявлено недовольство качеством изготовления (33%, 175/525), подгонки (57%, 297/525) и функциональностью протеза (26%, 139/525). В контрольном опросе 36% клиентов (115/321) сообщали о неудобстве или боли. Большинство клиентов (78%, 238/305) высказались в пользу протезов местного изготовления на будущее. Подавляющая часть клиентов (81%, 253/313) сообщали, что используют новое устройство не очень часто, редко или не пользуются им вовсе, при этом 88% клиентов (243/277) с имевшимся ранее устройством сообщали, что используют его часто или на постоянной основе. По меньшей мере 29% участников опроса (93/321) продолжили использовать предыдущее устройство, о котором ранее (во время пребывания во временном пункте) говорили, что тот протез сломан, не используется, плохо подогнан или причиняет боль.

**Вывод:**

Результаты опроса демонстрируют, что проблемы недостаточного качества и удовлетворенности в отношении исследованной системы протезирования остаются нерешенными и что многие клиенты полагаются на неподходящие или потенциально опасные для них протезы. Результаты также ставят на повестку дня новые вопросы подбора клиентов и эффективного использования финансирования в формате интенсивных программ на базе временных протезных пунктов.

## Introduction

Functional limb prostheses (artificial legs and arms) can greatly enhance the lives of individuals with amputation by improving mobility, fostering independence and enabling social participation.^1^^–^^4^ In low-resource settings, particularly those affected by humanitarian disasters, conflicts or landmine legacies, the unmet need for prosthetics and orthotics is disproportionately high.^5^^,^^6^

Short-term initiatives such as prosthetic and orthotic fitting camps aim to address such unmet need by providing devices to large numbers of individuals during intensive sessions, a similar principle to vaccination camps. These camps are distinct from mobile services operated by local prosthetic providers,^7^ as they are typically funded and delivered by nongovernmental organizations (NGOs) who often operate internationally. To ensure that recipients receive appropriate devices, providers need to maintain high standards in device design, fabrication quality and adequacy of aftercare, so as to prevent potential harm and avoid negative impacts on individuals and existing local services.^8^ The International Society for Prosthetics and Orthotics has expressed concern about short-term mission interventions,^8^ and their definition^9^ of appropriate technology in prosthetics and orthotics stresses that prosthetic rehabilitation services should ensure proper fit and alignment based on sound biomechanical principles, meet individual needs, and remain both affordable and sustainable with local resources. Notable examples of established devices include the polypropylene limbs introduced by the International Committee of the Red Cross^10^^–^^12^ and devices produced by the India-based NGO Jaipur Foot.^13^

Following extensive use of landmines by all parties to protracted conflicts between 1955 and 1979, Cambodia has become a compelling case study of positive results from international support for landmine clearance as well as physical rehabilitation and associated community services. Although the Cambodian government reportedly struggled to manage a multitude of other external health interventions,^14^ NGOs operating in the country have successfully established sustainable, locally-managed prosthetics and orthotics services.

Between the late 1980s and early 1990s, the original Jaipur foot prostheses were replaced with polypropylene prostheses from the International Committee of the Red Cross; around the same time, training from the International Society for Prosthetics and Orthotics and the World Health Organization (WHO) became available. The polypropylene prostheses are often used with domestically produced rubber feet which are well designed and tested; although somewhat heavy, these feet have excellent longevity, even if unprotected by a shoe.^15^ By 2023, Cambodia’s physical rehabilitation network comprised 11 centres run by the Cambodian government and international NGOs, all using standardized polypropylene prostheses. 

At the time of writing, Jaipur Foot reports having held 111 on-the-spot prosthetic fitting camps since 1975 across 44 countries in Africa, Asia and Central and South America.^16^ In March 2023, Jaipur Foot organized an intensive prosthetic limb-fitting camp near the city of Sisophon in Cambodia’s Banteay Meanchey Province, near the Cambodia–Thailand border. Following diplomatic discussions with the Indian Embassy, the Royal Government of Cambodia Mine Action and Victim Assistance Authority requested an independent assessment of the Jaipur foot prosthetic devices fitted by visiting technicians during the camp. In this paper, we report on this independent evaluation of the quality of the devices delivered as well as client satisfaction.

## Methods

We use the Strengthening the Reporting of Observational Studies in Epidemiology cohort study guidelines to report this study.^17^ Ethical approval for data collection was granted by the National Ethics Committee for Health Research in Cambodia (ref.160) and approval for analysis was granted by the University of Southampton Ethics and Research Governance Office (ref.102303).

### Study design

Our study design was tailored to assess outcomes and user experiences during and after an intensive fitting camp. We were permitted to conduct assessments but not to intervene, so our cohort study was purely observational.

We invited all clients attending the camp to participate. We evaluated service quality and client satisfaction using a two-stage approach. The first stage included a standardized assessment of device quality, completed jointly by a prosthetist and the client immediately after device fitting and discharge (that is, at device delivery). The second stage, 3 months later, involved a telephone interview with the client.

#### Stage 1: device delivery

International Society for Prosthetics and Orthotics-certified prosthetist–orthotist clinicians from the Cambodian School of Prosthetics and Orthotics completed a device assessment procedure and checklist, administered via a Microsoft Form (Microsoft, Redmond, United States of America). The assessment procedure and checklist were based on those used at device assessment in Cambodian physical rehabilitation centres (online repository),^18^ and reflect the professional standards used by the Cambodian School of Prosthetics and Orthotics to assess final-year prosthetist–orthotist students’ skills and readiness to graduate and gain International Society for Prosthetics and Orthotics certification. The checklist included 12 questions, evaluating five factors associated with device quality (quality of work, socket fit, height, dynamic gait and alignment, and residual limb condition); and seven factors related to client acceptance (comfort and stability, adequacy of suspension and straps, independent use, client’s needs, cosmesis [aesthetic appearance], device care, and satisfaction). Finally, the clinicians provided further comments on the assessment form, allowing a summary of their observations and any additional detailed comments made to them by the clients. Pairs of clinicians from a team of nine assessed device quality. Each pair included a senior prosthetist (more than 15 years’ experience) and a second prosthetist (minimum of 5 years’ experience). These assessors had no prior knowledge of the clients and were independent from the technicians that provided the devices.

#### Stage 2: follow-up interviews

Three months after device delivery, five of the prosthetists involved in stage 1 conducted follow-up telephone interviews with consenting, contactable clients. Interviews were semi-structured, with questions focused on the status and frequency of use of the new device, the ongoing use of any previous device, and client preferences for a future device. The 3-month follow-up period was chosen as sufficient for the client to get used to their new prosthesis.

### Data analysis

To maintain objectivity and reduce researcher bias, three authors who did not participate in data collection conducted the data analysis.

#### Stage 1: device delivery

We coded data from the positive and negative responses to the 12 checklist questions and analysed the data using descriptive statistics. We included categories for missing data. We analysed open-ended responses using content analysis,^19^ an established way to categorize text and summarize response frequencies. For coding, we treated each response as a separate unit and assigned a descriptive code based on content. We then organized codes into a coding frame which we used to analyse related responses; we added new codes if existing codes did not adequately capture the content. We used the final coding frame to systematically code the data and determine the frequency of responses. Two authors discussed and revised the coding process and tentative categories throughout the analysis and reached consensus with only minor modifications needed.

#### Stage 2: follow-up interviews

We coded data from the telephone follow-up interviews into categories representing device status, use level and preference. We then analysed data using descriptive statistics.

## Results

### Stage 1: device delivery

The team completed device delivery assessments for 532 individual clients who received a total of 542 devices. Data were collected for all except six clients who left the camp before they could be spoken to. We left nine orthotic devices out of the subsequent analysis, leaving 533 prosthetic limbs prescribed to 525 individual clients (Table 1).

**Table 1:** Client demographics, their health characteristics and details of previous prosthetic device(s), Cambodia, 2023

Characteristic	No. (%)
**Client demographics (*n* = 525)**	
Gender^a^	
Women	24 (9.0)
Men	243 (91.0)
Data missing^b^	258 (NA)
Age, in years	
0–29	3 (1.8)
30–39	8 (4.7)
40–49	8 (4.7)
50–59	68 (39.8)
60–69	70 (40.9)
≥ 70	14 (8.2)
Data missing^b^	354 (NA)
**Client health history (*n* = 525)**	
Client type	
Experienced (prosthesis user)	475 (91.2)
Primary (no previous device)	46 (8.8)
Data missing^c^	4 (NA)
Cause of amputation	
Landmine, weapon or ordnance	465 (89.9)
Electric shock	7 (1.4)
Traffic accident	19 (3.7)
Illness	5 (1.0)
Machine injury	12 (2.3)
Congenital	8 (1.5)
Other	1 (0.2)
Data missing^c^	8 (NA)
Year of amputation	
2020–2023	14 (2.8)
2010–2019	27 (5.3)
2000–2009	34 (6.7)
1990–1999	212 (42.0)
1980–1989	212 (42.0)
1970–1979	6 (1.2)
1960–1969	0 (0.0)
Data missing^c^	20 (NA)
No. of previous devices	
1	54 (11.1)
2–3	145 (29.8)
4–6	154 (31.6)
7–10	67 (13.8)
11–20 No previous device	21 (4.3) 46 (9.5)
Data missing^c^	38 (NA)
Condition of previous device	
Good	125 (24.0)
Condition unclear	138 (26.5)
Broken and/or damaged and/or worn	127 (24.4)
Old	30 (5.8)
Poorly fitting and/or painful	46 (8.8)
Stopped using	8 (1.5)
None (no previous device, lost, etc.)	47 (9.0)
Data missing^c^	4 (NA)
Time elapsed since receipt of previous device	
0–1 years	72 (14.7)
2–3 years	72 (14.7)
4–5 years	82 (16.7)
6–9 years	78 (15.9)
10–19 years	109 (22.2)
20 or more years	32 (6.5)
No previous device	46 (9.4)
Data missing^c^	34 (NA)
**Indication (*n* = 533)**	
Amputation level	
Partial foot	1 (0.2)
Ankle disarticulation	3 (0.6)
Transtibial	374 (70.2)
Knee disarticulation	12 (2.3)
Transfemoral	106 (19.9)
Wrist disarticulation	2 (0.4)
Transradial	34 (6.4)
Transhumeral	1 (0.2)
Side	
Right	252 (50.6)
Left	236 (47.4)
Bilateral	10 (2.0)
Data missing^c^	35 (NA)

NA: not applicable.

^a^ Gender was ascertained by the assessor.

^b^ High numbers of records were missing for gender and age because these were not collected in the digital study record; a partial record was replicated from paper notes.

^c^ Some responses were missing for individual clients as the form did not have mandatory fields.

Note: Inconsistencies arise in some values due to rounding. The numbers for the data missing category are excluded from the percentage calculation.

The median age was 59 years (interquartile range, IQR: 54–64). Clients were predominantly male, the most common amputation level was transtibial, and the most common cause of amputations was landmine or other weapon or ordnance injury. The median time since last device delivery was 5 years (IQR: 3–11). All except five clients consented to follow up.

**Fig. 1:**
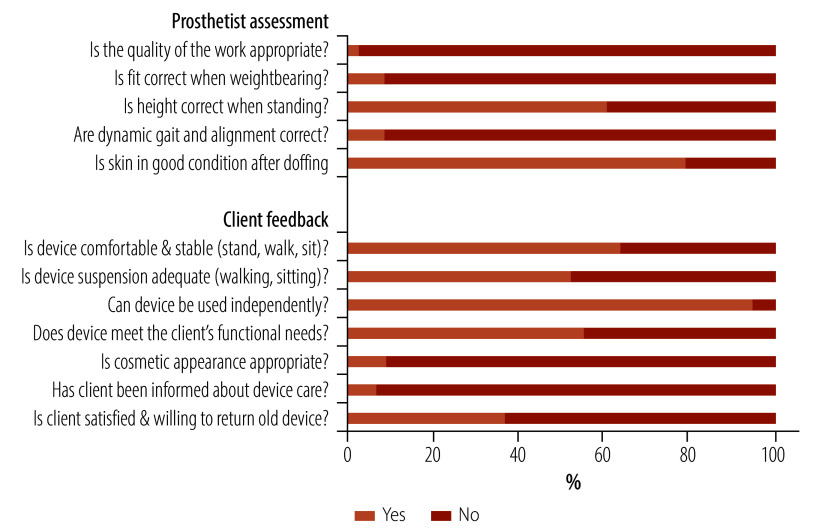
Prosthetist assessments of quality, and client feedback on satisfaction, immediately after camp discharge, Cambodia, 2023

Note: absolute numbers are available in Table 2.

**Fig. 2:**
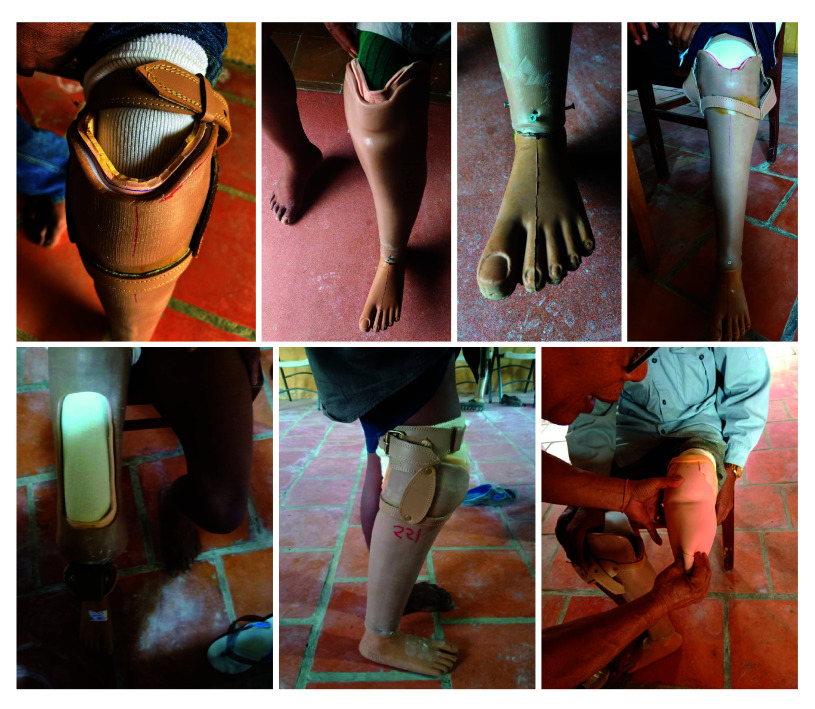
Photographs of representative example prosthetic devices showing poor device design and fabrication quality, Cambodia, 2023

Notes: top row, left to right: devices and liners were observed to have thick walls, with roughly finished brims. Some had exposed screws at the ankle, and poor cosmesis including pen marks, excess adhesive, and visible transition between cosmesis and foot. Bottom row, left to right: other devices had poor alignment of componentry such as knee-axis height, or the socket positioned in flexion, or poorly-sized liners and sockets.

Device delivery assessments (Fig. 1 and Table 2) identified several areas in which the new devices did not meet safety, quality of work and client satisfaction criteria. Examples of poor quality of work and cosmetic appearance are shown in Fig. 2. Overall, the clients’ opinion about the protheses was more positive than the prosthetists’ (Table 2). However, nearly two thirds of clients (286/453) who already had a device were not sufficiently satisfied with the new device to give up their previous device.

**Table 2:** Quality assessment by prosthetist y and client satisfaction feedback at the point of discharge from the camp, Cambodia, 2023

Question	No. (%)	
	Yes	No
**Prosthetist assessments**		
Is the general quality of work appropriate? (*n* = 522)	15 (2.9)	507 (97.1)
Is the socket fit of device correct? (weightbearing, total contact, shape, socket fitting, comfortable?) (*n* = 519)	45 (8.7)	474 (91.3)
Is the height of the device correct when the client is standing? (*n* = 518)	315 (60.8)	203 (39.2)
Are the dynamic gait and alignment correct when the client is walking? (pole vertical, foot full contact, stable, any major gait deviations?) (*n* = 506)	44 (8.7)	462 (91.3)
After doffing (removing) the device, is the client’s residual limb in good condition?^a ^(*n* = 514)	407 (79.2)	107 (20.8)
**Client feedback**		
While standing, walking and sitting is the device comfortable and stable? (*n* = 521)	333 (63.9)	188 (36.1)
While walking and sitting, is the suspension (or are the straps) adequate? (*n* = 520)	272 (52.3)	248 (47.7)
Can the patient use the device independently (e.g. during donning, doffing and walking)? (*n* = 522)	495 (94.8)	27 (5.2)
Does the device's function meet the patient needs? (*n* = 514)	285 (55.4)	229 (44.6)
Is the device's cosmetic appearance appropriate for the client’s needs? (*n* = 522)	47 (9.0)	475 (91.0)
Has the client been informed about device care? (*n* = 522)	35 (6.7)	487 (93.3)
Is the client satisfied with new device and willing to give up their current device? (*n* = 453)	167 (36.9)	286 (63.1)

^a^ Pressure on the number of clients to see at the camp within the study period meant that residual limb condition was assessed after approximately ten minutes of device use; this time may be insufficient to detect risk of residual limb injury. In a conventional clinic, residual limb injury is usually assessed after approximately one hour of device use (i.e. gait training).

### Analysis of open-ended comments

Content analysis of free-text responses made by clinicians at device delivery in the open-ended section of the assessment form (Table 3) revealed a wide range of reasons for client dissatisfaction and highlighted inadequacies associated with use and mobility, cosmesis and finish, device fit and client experience. Such inadequacies are consistent with the quality assessment of devices and photographic evidence (Fig. 2). Clinicians reported gait deviations (139/525; 26%), wrong height (110/525; 21%) and poor alignment (66/525; 13%). The majority also reported observing, or the client reporting, a socket fit that was either too loose (230/525; 44%) or too tight or high pressure (67/525; 13%); and with poor finishing (175/525; 33%), for example, with pen marks, exposed rivets, sharp trim lines and unadhered rubber cosmeses. Nevertheless, the clinicians reported that some clients commented positively on the device’s light weight (51/525; 10%; Table 3).

**Table 3:** Content analysis of free text comments at prosthetic device delivery, Cambodia, 2023

Category and code	No. of responses
**Use and mobility**	
Client can walk, do activities of daily living	4
Gait deviation	139
Prosthesis wrong height	110
Poor alignment	66
Difficulty donning and doffing	14
Difficult or unstable walking	12
Knee functions poorly, or hard to control	12
Noise when walking	6
Difficulty wearing device	5
Requires training or practice	5
Cannot walk unaided	3
Walks with knee locked	2
Cannot walk at all	1
**Cosmesis and finish**	
Client likes appearance	2
Poor cosmesis or finish in general	175
Trim line sharp or uneven	74
Cuff suspension visible through long pants	5
Rivet or cuff not secure	2
Socket attachment broken or loose	2
Visible when sitting	2
Rivet showing	1
Rubber not stuck (unadhered cosmesis)	1
Prosthesis not strong enough	1
**Socket fit**	
Good socket fit in general	19
Too loose	230
Poor socket fit in general	143
Too tight, or high pressure	67
Incorrect trim line height	29
Inadequate suspension	10
**Experience**	
Client likes light weight	51
Socket causes pain	19
Socket not smooth	10
Prosthesis uncomfortable	6
Prosthesis too heavy or bulky	2
Straps too tight	1

Note: the content analysis include the device delivery assessments for 533 prosthetic limbs prescribed to 525 individual clients**.**

### Stage 2: follow-up interviews

Between 25 May 2023 and 12 June 2023, 327 clients were contacted and completed a follow-up telephone interview. Clients that did not answer the telephone despite multiple attempts, or that had telephone numbers that were found to be incorrect, were considered as lost to follow-up. Responses for six clients who were orthosis users were set aside, leaving 321 prosthesis user clients followed up.

Analysis of interview questions at the follow-up, excluding missing data, showed that a total of 140 (44%) clients considered their new device comfortable or somewhat comfortable, compared to 115 (36%) not comfortable or painful (Table 4 and online repository).^18^ Six reported that the new device was broken.

**Table 4:** Client feedback on prosthetic device condition, use level and preference during a 3-month telephone follow-up, Cambodia, 2023

Question	No. (%)
**What is the condition of the new device?**	
Comfortable	107 (33.3)
Somewhat comfortable	33 (10.3)
Not comfortable, or painful	115 (35.8)
Broken	6 (1.9)
Does not use	55 (17.1)
Other	5 (1.6)
**If you can compare, which device can you walk further and faster on?^a^**	
New device	35 (12.0)
Previous device	236 (80.8)
Neither, or the same	21 (7.2)
Cannot compare	29 (NA)
**In the future, which device would you prefer to have?^a^**	
Another like the new device	47 (15.4)
A locally-produced device	238 (78.0)
No preference, or the same	20 (6.6)
Cannot compare	5 (NA)
Data missing	11 (NA)
**What is the use level of the new device?^b^**	
Always	60 (19.2)
Often	0 (0.0)
Short periods	125 (39.9)
Rarely	95 (30.4)
Never or cannot	33 (10.5)
Data missing	8 (NA)
**What is the use level of the previous device?^b^**	
Always	180 (65.0)
Often	63 (22.7)
Short periods	1 (0.4)
Rarely	15 (5.4)
Never or cannot	6 (2.2)
Broken	12 (4.3)
Does not have	40 (NA)
Data missing	4 (NA)

NA: not applicable.

^a^ Excludes those that cannot compare devices, such as primary patients, as they do not have a previous device.

^b^ Use level categories represent pooled responses: never or cannot (“never” or “cannot use”); rarely (“rarely” or “almost never”); short periods (“short periods every day” or “short periods some days” or “not very often”); often (“often” or “most of the time”); always (“always” or “all day”).

Note: the numbers for the “data missing”, “cannot compare”, or “does not have categories” are excluded from the percentage calculation.

Of the clients able to compare between new and previous devices, 12% (35/292) said they could walk further and faster with the new device compared to 81% (236/292) who selected their previous device, while 7% (21/292) reported both devices were similar (Table 4 and online repository).^18^ Most clients who could compare said they would prefer a locally-produced device in future (78%; 238/305).

**Fig. 3:**
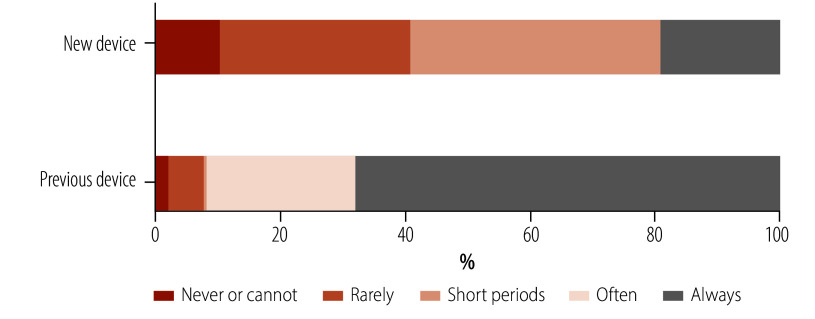
Client report of new and previous prosthetic device usage at 3-month follow-up, Cambodia, 2023

Note: absolute numbers are available in Table 4. Use level categories represent pooled responses: never or cannot (“never” or “cannot use”); rarely (“rarely” or “almost never”); short periods (“short periods every day” or “short periods some days” or “not very often”); often (“often” or “most of the time”); always (“always” or “all day”).

**Fig. 4:**
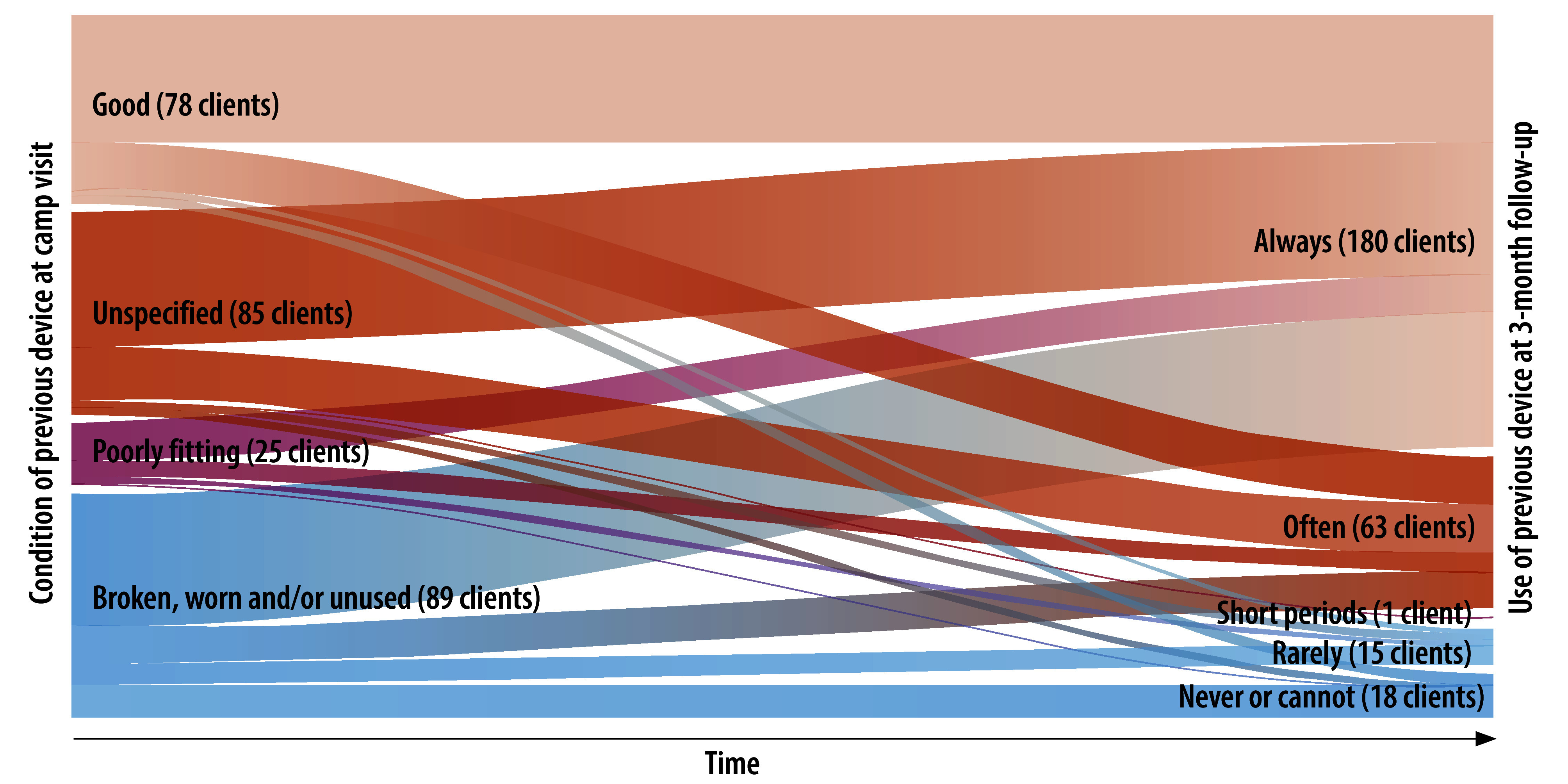
Mapping from the clients’ reported previous prosthetic device condition to their level of use of their previous device at 3-month follow-up, Cambodia, 2023

Note: condition categories represent pooled responses: good (“good” or “ok”); unspecified (“cannot determine” or “condition not specified”); poorly fitting (“painful” or “tight” or “loose”); broken, worn and/or unused (“broken” or “damaged” or “worn out” or “needs repair” or “old or “stopped using”). Use level categories represent pooled responses: never or cannot (“never” or “cannot use”); rarely (“rarely” or “almost never”); short periods (“short periods every day” or “short periods some days” or “not very often”); often (“often” or “most of the time”); always (“always” or “all day”).

Finally, we assessed clients’ usage of both the new and previous prosthetic devices at 3-month follow-up (Table 4 and Fig. 3). Most respondents (253/313; 81%) reported never, almost never or rarely using their new device, or using it for short periods, some days, or not very often; while 60/313 (19%) reported always using their new device. Asked about their previous device, of clients who still had one, 243/277 (88%) reported using it always, often or most of the time. A high proportion who had a previous device at the camp visit had reverted to using it always, often or most of the time across all previous device condition categories (Fig. 4). Of clients whose previous device was damaged or worn out, only 28% (27/96) were using their new device all the time.

## Discussion

Our study was conducted at the request of the Royal Government of Cambodia’s Mine Action and Victim Assistance Authority following the organization of a camp providing Jaipur foot devices. We found that the outcomes did not meet International Society for Prosthetics and Orthotics benchmarks, which include 95% prosthesis use, 90% satisfaction, 60% good socket fit, 90% acceptable alignment and 90% good quality of fabrication^20^^,^^21^ In conventional Cambodian physical rehabilitation centres, devices failing any of the 12 quality or satisfaction checks assessed in this study at delivery would be reworked, especially when failures might lead to poor function or potential injury, for example by modifying to correct the device’s fit, length or alignment. Additionally, the outcomes were less favourable than those reported in multiple other low-resource settings.^15^

While the Jaipur foot devices used in fitting camps meet many mechanical, social and cultural-specific needs, they have faced criticism for their weight, lack of manufacturing standardization and limited durability, particularly for individuals with higher body weights.^22^^,^^23^ Our findings suggest low quality and low client satisfaction remain key concerns for these devices, aligning with reports from more than 20 years ago when International Society for Prosthetics and Orthotics-affiliated authors conducted a 3-country, approximately 3-year follow-up of these devices for transtibial^24^ and transfemoral^25^ amputations. They identified poor quality of fabrication in 56% of transtibial and 86% of transfemoral devices, primarily poor fit, alignment and socket wall adequacy, and discrepancies in the length between the two legs. Although satisfaction and compliance were reported to be good for transtibial device users (85% and 94%, respectively) and moderate in transfemoral device users (58% and 65%, respectively) fewer than half the participants could walk more than 1 kilometre and many reported discomfort and pain. While these papers^24^^,^^25^ reported a considerably longer follow-up than in our study, the device fit, length and alignment shortcomings we found at 3 months cannot be expected to improve without further intervention. It is important to note that measures to manage inadequate fit, such as accommodating socket looseness by wearing socks, would likely be undesirable in the warm, humid climate experienced for most of the year in Cambodia.^24^

We found that many clients retained their old devices, with a large proportion reverting to them within 3 months of receiving their new prosthesis. This observation suggests that, despite initially more positive assessments about the new devices, over time, clients’ experience aligned with prosthetists’ assessments at fitting. We found that clients expressed some satisfaction with their new devices despite noting discomfort, imperfect fit and concerns about cosmesis and quality of work. Such deference may reflect cultural norms in Cambodia,^26^ with clients being unaccustomed or reluctant to voice concerns about their physical rehabilitation care and prosthetic devices. Such behaviour may be more prominent in rural communities, especially when services are provided free of charge.^27^

Our findings also raise questions about client selection, specifically whether camp attendees needed a new device. Camp organizers recruited clients for the camp with the assistance of local authorities but had no insights into clinical need. Previous analysis of a NGO-established and locally-run physical rehabilitation service in Cambodia showed a median time for device repair of 2 years.^12^ In our study, clients reported using previous devices for a median of 5 years, suggesting a notable proportion of clients had poorer access to physical rehabilitation services than those served by conventional physical rehabilitation centres in the previous study. However, we also found a quarter of clients had a previous device deemed still in good condition at the point of new device delivery. Some previous devices were delivered within the last year, with some devices being less than 3 months old. At the same time, our findings also suggest a large number of clients were tolerating an inadequate or potentially dangerous prosthesis. Although 209 clients had a previous device reported as unused, broken, painful or poorly fitting, at least 93 of them had reverted to using these devices often, most or all of the time within three months. Notably, both the percentages of clients who had a well-functioning alternative device before the camp (indicating poor client selection), and those who had returned to using an inadequate device 3 months after it (indicating poor device quality), are likely to be underestimates, given that a quarter of clients had a previous device whose condition could not be determined from the responses.

Our study has some limitations. We used an assessment checklist which considered quality and satisfaction against criteria previously defined by the International Society for Prosthetics and Orthotics. This checklist was not previously standardized or validated; nevertheless, it reflects the device delivery assessment procedure used as part of the ISO 9001 quality management system at established Cambodian physical rehabilitation centres for over 20 years.^18^

We found that using the checklist in the field led to some heterogeneity in completion of questions, and some potentially valuable client data, such as gender, were not captured. In addition, errors and occasional data conflicts might have arisen due to needing to assess all clients and collect large amounts of data in a timely manner. The relatively large number of assessors collecting data potentially increased the likelihood of inter-assessor subjectivity; notably, our protocol did not include a process of recording or cross-checking interview transcripts. About two fifths of the study participants could not be contacted for the follow-stage 2 telephone interviews and were considered lost to follow-up. However, no substantial differences were observed between the stage 1 data for the clients lost to follow-up and the full group (online repository),^18^ indicating no systematic link between satisfaction or quality and loss to follow-up.

Co-inventor of the Jaipur foot, Dr Sethi, stated his vision was to increase prosthetic knowledge and simplify technology, with camps not only providing devices but also training local artisans to fabricate, adjust and repair them.^13^ Despite the large number of camps and delivered devices reported,^16^ it has been stated that “[s]omewhere down the line, the number of amputees fitted at these camps overtook the concept of imparting training to the local artisans, and it all boiled down to a game of numbers.”^28^ This situation has been attributed in part to replacement of aluminium sockets with thermoplastics. This change made fabrication quicker and cheaper, but the thermoplastic prosthetics are heavier, and it is more difficult to achieve the desired alignment and fit. An International Society for Prosthetics and Orthotics-affiliated follow-up study of these devices in 2004^25^ reported that while “material and components are of high technical standard and could provide a low-cost possibility…the untrained, so-called technicians are unable to adapt a prosthesis to an amputation stump.” That study also stated that “recognized prosthetics training is required to ensure proper use of materials and correct alignment of the prosthesis.” We offered the camp organizers both a training needs analysis and training for technicians working in the camp, but this proposal was not accepted.

Although we cannot confirm generalizability, our study suggests that previous concerns regarding quality and satisfaction with the studied prosthesis system may persist. Despite the camp organizers' best intentions to provide care to those who may not be able to access it, our study also presents new evidence that questions whether provision of prosthetic limbs at intensive limb-fitting camps represents an inclusive model of care or an effective use of funding. Previous research has shown that device durability and access to repairs and servicing are reported as issues of top priority to people in low-resource settings who use prostheses and orthoses.^4^^,^^29^^,^^30^ Inadequate training of people designing, fabricating and fitting devices, and inadequate follow-up care may burden local physical rehabilitation services (where they exist), or leave vulnerable clients without support, especially as the camp format and devices delivered do not integrate with the currently available services.

Building on our findings, we suggest that camps may be more appropriate for clients whose need is clearly demonstrated, should be fully integrated with existing services, and should leave behind adequate materials and components for repairs and replacement. Screening clients for need is an essential prerequisite, as is engagement with the local practitioners who will be expected to continue client care.
